# P2X7 receptor-activated microglia in cortex is critical for sleep disorder under neuropathic pain

**DOI:** 10.3389/fnins.2023.1095718

**Published:** 2023-02-03

**Authors:** Tingting Li, Yunling Gao, Mengying He, Zhu Gui, Bingchu Zhao, Yue Cao, Ting Chen, Jinpiao Zhu, Jie Wang, Qi Zhong, Zongze Zhang

**Affiliations:** ^1^Department of Anesthesiology, Zhongnan Hospital, Wuhan University, Wuhan, Hubei, China; ^2^Xiangyang Central Hospital, Institute of Neuroscience and Brain Diseases, Affiliated Hospital of Hubei University of Arts and Science, Xiangyang, Hubei, China; ^3^Key Laboratory of Magnetic Resonance in Biological Systems, State Key Laboratory of Magnetic Resonance and Atomic and Molecular Physics, National Center for Magnetic Resonance in Wuhan, Wuhan Institute of Physics and Mathematics, Innovation Academy for Precision Measurement Science and Technology, Chinese Academy of Sciences, Wuhan National Laboratory for Optoelectronics, Wuhan, China; ^4^University of Chinese Academy of Sciences, Beijing, China; ^5^School of Computer Science, Wuhan University, Wuhan, Hubei, China

**Keywords:** P2X7R, neuropathic pain, microglia, sleep disturbance, thalamocortical oscillation

## Abstract

Neuropathic pain (NP) is associated with sleep disturbances, which may substantially influence the quality of life. Clinical and animal studies demonstrated that neurotransmitter is one of the main contributors to cause sleep disturbances induced by NP. Recently, it was reported that P2X7 receptors (P2X7R) are widely expressed in microglia, which serves crucial role in neuronal activity in the pain and sleep-awake cycle. In this study, we adopted the chronic constriction injury (CCI) model to establish the progress of chronic pain and investigated whether P2X7R of microglia in cortex played a critical role in sleep disturbance induced by NP. At electroencephalogram (EEG) level, sleep disturbance was observed in mice treated with CCI as they exhibited mechanical and thermal hypersensitivity, and inhibition of P2X7R ameliorated these changes. We showed a dramatic high level of P2X7R and Iba-1 co-expression in the cortical region, and the inhibition of P2X7R also adversely affected it. Furthermore, the power of LFPs in ventral posterior nucleus (VP) and primary somatosensory cortex (S1) which changed in the CCI group was adverse after the inhibition of P2X7R. Furthermore, inhibition of P2X7R also decreased the VP-S1 coherence which increased in CCI group. Nuclear magnetic resonance demonstrated inhibition of P2X7R decreased glutamate (Glu) levels in thalamic and cortical regions which were significantly increased in the CCI mice. Our findings provide evidence that NP has a critical effect on neuronal activity linked to sleep and may built up a new target for the development of sleep disturbances under chronic pain conditions.

## 1. Introduction

Chronic neuropathic pain (NP) is a prevalent health problem, affecting approximately 10% of the general population (Scholz et al., 2019). Sleep disturbance is another one of the most frequent problems for the adults with chronic pain, and a previous study estimated that 68% of chronic NP patients have poor sleep quality (Lampl et al., 2010). These mild sleep disturbances could increase the risk of developing fatigue, harm mood, cognitive function, injured tissues, and impaired brain function (Sexton et al., 2020; O’Gara et al., 2021). Therefore, a better knowledge of the physiological mechanisms underlying sleep disturbance induced by chronic pain is necessary for counteracting the cognitive dysfunction in later life.

To explore and validate the adverse effects of chronic NP, several animal models have been developed to imitate the peripheral nerve injury, of which the most frequently used is chronic-constriction injury (CCI) model (Austin et al., 2012; Wang H.-R. et al., 2021). The evidence highlighted chronic NP activated a widespread neural network consisting of the primary somatosensory cortex (S1) region of parietal cortex (PC), and the ventral posterior nucleus (VP) region of the thalamus (TH) (Witting et al., 2001; Seifert et al., 2013; Kim et al., 2017; Asseyer et al., 2020). The thalamus served as a key relay station for transmitting nociceptive information to the cerebral cortex after CCI, moreover, recent studies had shown that thalamocortical connections participated in several aspects of sleep-wake states (Gent et al., 2018). Hitherto, the investigations had emphasized the role of brain rhythmic oscillatory activity in NP development (Kim and Davis, 2021). Nevertheless, whether the thalamocortical area contribute to the sleep disturbance induced by NP remains an open question and stands as a future challenge for basic science and healthcare research.

The P2X7 purinoceptors are widely present in glial cells and neurons of CNS (Deng et al., 2021), and they are dependent on ATP ligand-gated ion channels (Andrejew et al., 2020). Early reports of antagonists and KO mice have suggested that the microglial P2X7R was involved in chronic NP (Fulgenzi et al., 2008), and P2X7R is upregulated in the spinal cord after nerve injury (Tsuda, 2017). In addition, P2X7R-knockout mice fail to develop behavioral hypersensitivity after partial sciatic nerve ligation (Kambur et al., 2018). When the P2X7 receptor bounds to ATP, it can open the ion channel on the cell membrane, lead to sodium and calcium ion influx and potassium ion outflow, which could upregulate the P2X7 expressions in glial cells and neurons, increase the potential activities of the cell membrane, and sensitize the nerve system, eventually inducing the occurrence of pain (Zhang et al., 2020). Moreover, P2X7R was implicated in sleep regulation (Metzger et al., 2017), brain levels of P2X7R change with sleep loss and time of day. Additionally, P2X7R expression is increased following sleep deprivation in humans. Furthermore, P2X7R directly modulates the action of somnogenic cytokines including IL-1β and tumor necrosis factor α, which are recognized as endogenous sleep regulatory substances, and mice with P2X7R variants showed the alterations in their sleep quality (Metzger et al., 2017). Furthermore, morphological and functional variations of cortical microglia was observed in the sleep-wake cycle (Garofalo et al., 2020). Indeed, recent work demonstrated that cortical microglia possessed an intrinsic molecular clock (Fonken et al., 2015) and played a key role in sleep disturbance (Xiao et al., 2022). Mice lacking the microglial P2X7 receptor have attenuated sleep rebound responses following sleep loss (Krueger et al., 2011). However, whether microglial P2X7R in cortex contributes to the sleep disturbance induced by NP is unknown.

Previous studies have demonstrated that sleep disturbance induced by NP changes the expression of neurotransmission (Ito et al., 2020). A growing amount of evidence indicated that the P2X7R carried out slow neuromodulatory function, speeded up synaptic transmission and neurotransmitter release in the central and peripheral nervous system (Miras-Portugal et al., 2017). Moreover, many experimental studies have demonstrated that P2X7R can alter neuronal activities (Chen et al., 2017), which may result from the change of neurotransmitter. Therefore, we postulated that microglial P2X7R in cortex contributed to neural oscillations *via* neurotransmitter modulation in sleep disturbance induced by NP.

In the present study, we established a chronic-constriction injury model of mice and applied the P2X7R inhibitor. Here, we combined animal behaviors, molecular approaches, EEG and LFP recording, metabolic features to further understand our hypothesis. Thus, the current study tried to disclose the roles of microglia P2X7R in cortex on the NP-induced sleep disturbance, which should be necessary for the future clinical study.

## 2. Materials and methods

### 2.1. Animals

**FIGURE 1:**
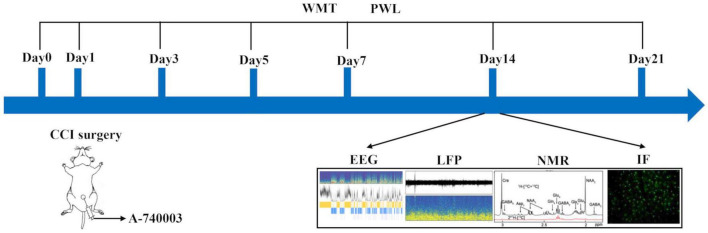
Illustration of the experimental design. Four different groups were involved. (1) Sham, the Sham group; (2) Sham + A-740003, the Sham mice treated with A-740003; (3) CCI, the chronic-constriction injury group; (4) CCI + A-740003, the CCI mice treated with A-740003. The model continued for 21 days. Mice were subjected to investigate pain hyperalgesia by the mechanical paw withdrawal threshold (MWT) and paw thermal withdrawal latency (PWL) on day 0, 3, 5, 7, 14, 21; sleep states by EEG/EMG on day 14; local field potential (LFP) recordings on day 14; the metabolic kinetics study by ^1^H-NMR on day 14; and the immunohistochemistry for double P2X7R and microglia in the parietal cortex on day 14.

The entire animal experimental procedures were carried out in compliance with the NIH guidelines for the care and use of laboratory animals. The animal study protocol was approved by the Experimental Animal Welfare Ethics Committee of Zhongnan Hospital of Wuhan University (NO: ZN2021212, 29 November 2021). The adult male C57BL/6 wild-type mice (8–10 weeks; 25 ± 2 g) were obtained from the Experimental Animal Center of School of Medicine, Wuhan University. The animals were housed at 23 ± 2°C with a 12-h light/dark cycle and free access to water and food. To alleviate the stress of the animals to human interaction, the animals were handled daily until the experimental day, and the experimental flowchart is illustrated in Figure 1.

### 2.2. Chronic-constriction injury (CCI) model

The CCI animal model was developed following the previous description (Wang Y. Q. et al., 2021). Here, only a brief description was provided. Under sodium pentobarbital (40 mg/kg, *i.p.*) anesthesia, the right sciatic nerve was exposed and loosely ligated with 4–0 silk at four sites with an interval of 1 mm on the lateral side of the right femur after blunt dissection of the biceps femoris muscle under the skin incision. The tightness of the ligation should not affect the blood supply to the epineurium, nonetheless, the ligation could cause a minor tremor of the calf muscles. For the sham group, all procedures are completely identical to the CCI group, except for the nerve ligation. Finally, the incisions were separately closed and sutured using 3–0 silk.

Mice were divided into four groups at random: (1) Sham group: received 0.5 mL DMSO (*i.p.*; Aspen, AS1025, Wuhan, China); (2) Sham + A-740003 group: received a selective P2X7R antagonist—A-740003 [N-(1-{[(Cyanoimino) (5-quinolinylamino)methyl] amino} –2,2-dimethylpropyl)-2-(3,4-dimethoxyphenyl) acetamide] (*i.p.*; 0.5 mL, 180 mg/kg, dissolved in 1% DMSO, 44% PEG-300, 5.5% Tween-80, and 49.5% saline) (A0862, Sigma-Aldrich, Germany); PEG-300 has good compatibility with many organic components and can be used to increase the solubility of A-740003. (3) CCI group: CCI model and received 0.5 mL DMSO (*i.p.*). (4) CCI + A-740003 group: CCI model and received A-740003 (*i.p.*; 0.5 mL, 180 mg/kg). Mice were given A-740003/DMSO daily for 3 days prior to the measurement.

### 2.3. Behavioral test

As previously described (Jiang et al., 2021), pain-related behavior indicators were detected. The mechanical paw withdrawal threshold (MWT) and the paw thermal withdrawal latency (PWL) were measured 1 day before the surgery, as well as on days 3rd, 5th, 7th, 14th, and 21st post-surgery. Briefly, the animals were exposed to the environment at least 15 min before the test. The *von Frey* fibers (IITC life, USA) were used to vertically stimulate the plantar surface of the ipsilateral hind paw and sustained for 5 s to evaluate MWT. When the stimulated paw was retracted, licked, or bit, the fiber strength was measured. Furthermore, the thermal radiation stimulation of a 390G bolometer (Shanghai Yuyan Scientific Instrument Co., Ltd.) was used to test the latency of withdrawal reflex to evaluate PWL. Briefly, the thermal source under a glass plate was targeted at the ipsilateral hind paw, and the time was recorded until the stimulated paw was abruptly withdrawn.

### 2.4. Microelectrode implantation

After anesthesia with pentobarbital sodium (40 mg/kg, *i.p.*), the mice were fixed on a stereotaxic apparatus (DW-2000, Techman, Chengdu, China), and the skull was completely exposed through a small incision around the middle line on the 7th day after injury. After the bregma was identified and marked, the microwire electrodes were epidurally implanted in the ventral posterior nucleus of the thalamus (VP) and primary somatosensory cortex (S1) from the left hemisphere using the proper coordinates (VP: AP + 1.82 mm; ML −1.70 mm; DV, −3.62 mm; and S1: AP + 3.00 mm; ML −2.06 mm; DV, −1.25 mm). The local field potential signals were recorded using these microwire electrodes. As previously described (Pal et al., 2011; Clasadonte et al., 2014), two stainless steel cranial nails were affixed to the skull within the frontal cortex using the proper coordinates (AP + 1.98 mm; ML −0.50 mm; and AP + 1.98 mm; ML + 0.50 mm) as the electroencephalogram (EEG) recording electrode and the reference electrode, respectively. Another steel cranial nail was implanted into the skull surfaces of parietal cortex (AP −2.0 mm; ML + 0.50 mm) and used as ground electrode. Finally, these nails were embedded with dental acrylic cement. The electromyogram (EMG) electrodes were implanted in the neck muscles. The animals were given a week to heal from the implantation surgery prior to the studies.

### 2.5. EEG and LFP recording and analysis

As our behavioral results showed, mechanical hyperalgesia and heat allodynia reaction began on the third day, reached the peak on the 14th day, and lasted on the 21st day after chronic-constriction injury. Thus, the day 14th, as the time when pain developed to the peak, has been chosen as the recording day. The animals were allowed to acclimate to their surroundings about 30 min before each test. The recorded EEG, EMG, and LFP signals were amplified by a 16-channel bioelectrical signal amplifier (Dagan Company of the United States), collected by the acquisition card micro_1401 (CED company of British), filtered at 50 Hz, and recorded in a processing system (Spike 2). The sample rate was set to 1,000 Hz. The states of wakefulness (WAKE), Non-rapid eye movement sleep (NREM), and Rapid eye movement sleep (REM) were identified by a combination of EEG, EMG, and behavioral criteria. The awake state was characterized by low amplitude, high-frequency beta wave activity, and high-amplitude EMG, accompanied by standing and moving around the cage with open eyes; NREM state was characterized by low frequency, high-amplitude delta wave activity, and low-amplitude EMG, accompanied by immobile posture with closed eyelids; REM state was characterized by low amplitude and high-frequency theta wave activity and the absence of muscular activity with the head totally relaxed.

The sleep stages of the recordings are scored by an AI-driven software, Lunion Stage, which is developed by LunionData (Shanghai Lunion Intelligent Technology Co., Ltd., China). A sleep episode was defined as any single 4-s epoch. When the recordings completed, state was classified automatically under 4-s epochs into three stages (wake, REM, NREM) using this software according to standard criteria above. The LFP waveform was converted into a power spectral histogram using the Fast Fourier transform function (FFT) in MATLAB (2020b) to plot the power distribution of each frequency band over time. According to the frequency, oscillations were classified as delta (1–4 Hz), theta (4–8 Hz), alpha (8–14 Hz), beta (14–30 Hz), and gamma (30–100 Hz) oscillations. We computed the power percentage of a certain frequency band by dividing the sum of the power values within that band by the total power between 1 and 100 Hz. To determine the spectral coupling among signals from VP and S1 recorded regions, the “cohere ultimate” script in Spike 2 was utilized to quantify the power transfer. The function returns a coherence value between 0 and 1, with a value of 1 signifying an exact match in the amplitude difference between the two waveforms.

### 2.6. Immunofluorescence

Mice were anesthetized with sodium pentobarbital (40 mg/kg, *i.p.*) and intracardially perfused with phosphate-buffered saline (PBS) and 4% cold paraformaldehyde on the 14th day after injury. The mouse brain was collected and dehydrated overnight in 30% sucrose solutions before being sectioned for immunofluorescence. Tissues were sectioned into 20-μm slices using a freezing microtome (Leica). The brain slices were washed and blocked for 60 min at 37°C in the blocking solution [5% donkey serum (Booster, China) diluted in PBS]. The slices were incubated overnight at 4°C with the following primary antibodies: P2X7R (1:200, rabbit, APR-004, Alomone, USA) and Iba-1 (1:200, goat, 011-27991, Wako, UK). Then, they were then washed and incubated with appropriate fluorescent secondary antibodies (Cy3 donkey anti-rabbit, 1:200; 711-165-152; Jackson; FITC donkey anti-goat, 1:200, 705-545-147, Jackson) for 1 h at room temperature. Finally, the slices were incubated with DAPI for 10 min. The fluorescent images were acquired using an Aperio VERSA 8 microscope (Leica, Germany).

### 2.7. Metabolic kinetics study

#### 2.7.1. Brain tissue collection

As aforementioned (Guo et al., 2022), all mice were fasted overnight and given only free access to water prior to the experiment day for decreasing the levels of endogenous glucose. On the 14th day after injury, the animal was initially anesthetized with 4.0% isoflurane, and a PE50 tube (Intech, PA, USA) was inserted into the lateral tail vein and immobilized. Then the animal was rehabilitated until they were free moving in its home cage. After 20 min, [1-^13^C] glucose (Qingdao Tenglong Weibo Technology Co., LTD., Qingdao, China) was infused through the tail vein catheterization at the rate of 400–600 μL/min in 2 min, and the dosage was computed based on the animal weight (Guo et al., 2021). After 30 min, the head centered on microwave irradiation technology was employed to euthanize the animals after deep anesthesia (5.0% isoflurane) with a specialized microwave apparatus (Tangshan Nano source Microwave Thermal Instrument Manufacturing Co., Ltd., China). The brain tissues, including the thalamus and parietal cortex, were isolated, weighed and immediately frozen at −80°C for future operations.

#### 2.7.2. Sample extraction

A total of 400 μL HCl/methanol (0.1 M) were added to brain tissue samples, and the mixture was homogenized for 90 s (frequency is 20 Hz). The samples were then mixed with 800 μL ethanol (60%, v/v) and homogenized under the same conditions. After that, the mixture was centrifuged for 15 min (4°C, 14, 000 *g*), and the supernatants were collected. This process was performed twice to collect the supernatant. All supernatants were lyophilized by a frozen vacuum drying machine (Thermo Scientific, Germany) after extracting the organic solvents with a normal vacuum machine at 45°C. The lyophilized samples were dissolved in phosphate buffer solution (600 μL of D_2_O with 0.2 M Na_2_HPO_4_/NaH_2_PO_4_, pH = 7.2), the TMSP [3-(trimethylsilyl) propionic-2,2,3,3-d_4_ acid sodium salt] was used as the internal standard in the buffer.

#### 2.7.3. [^1^H-^13^C]-NMR spectroscopy

All samples were placed in a BrukerAvance III 500 MHz NMR spectrometer (BrukerBioSpin, Germany) for ^1^H-NMR spectra acquisition, and the pulse sequence of POCE (^1^H-Observed/^13^C-Edited) was utilized to assess the metabolite concentrations and the ^13^C enrichments of the metabolites. The ^13^C enrichment of the metabolites was calculated from the difference of the two spectra in the POCE NMR spectrum. During the NMR spectrum processing, the baseline and phase of the NMR spectra were manually adjusted in the commercial software Topspin (Bruker BioSpin, Germany). Then, the adjusted NMR spectra were processed with a homemade software—NMRSpec (Liu et al., 2017), which is free available by request to jie.wang@wipm.ac.cn. The ^13^C enrichment of the specific metabolites (e.g., Glu_3_, Gln_4_, and Glx_3_) was automatically integrated and calculated in this software.

### 2.8. Statistical analysis

The statistical analyses were performed with GraphPad Prism version 8 and SPSS 26.0 statistical software. Statistical data evaluation was performed with a one−way analysis of variance (ANOVA), followed by Fisher’s least significant difference (LSD). *p* < 0.05 was considered as the statistically significant, and the results were presented as means ± SEM.

## 3. Results

### 3.1. Inhibition of P2X7R alleviated chronic-constriction injury induced pain hyperalgesia

**FIGURE 2:**
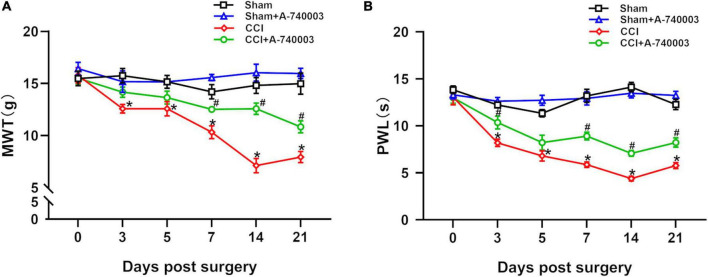
Inhibition of P2X7R alleviated chronic-constriction injury induced pain hyperalgesia. All mice groups were tested in mechanical paw withdrawal threshold (MWT) **(A)** and paw thermal withdrawal latency (PWL) **(B)** on 1 day before the surgery, 3rd, 5th, 7th, 14th, and 21st day post-surgery. Data are presented as mean ± SEM (*n* = 12). *p < 0.05, vs. Sham group, ^#^*p* < 0.05 vs. CCI group. Sham, the Sham group; Sham + A-740003, the Sham mice treated with A-740003; CCI, the chronic-constriction injury group; CCI + A-740003, the CCI mice treated with A-740003.

Here, MWT and PWL were utilized to assess the painful sensitivity of mice on 1 day before the surgery, 3rd, 5th, 7th, 14th, and 21st day post-surgery. The results of mechanical hyperalgesia and heat allodynia were collected in Figures 2A, B. Compared with the sham group, both mechanical hyperalgesia (12.6 g vs. 15.7 g on day 3, 12.6 g vs. 15.2 g on day 5, 10.3 g vs. 14.2 g on day 7, 7.1 g vs. 14.8 g on day 14, 7.9 g vs. 15.0 g on day 21) and heat allodynia (8 s vs. 12 s on day 3, 7 s vs. 11 s on day 5, 6 s vs. 13 s on day 7, 4 s vs. 14 s on day 14, 6 s vs. 12 s on day 21) significantly decreased in the CCI group from the third day after the surgery until 3 weeks after the surgery (*p* < 0.05). The A-740003 treatment attenuated the CCI-induced mechanical hyperalgesia (12.5 g vs. 10.3 g on day 7, 12.6 g vs. 7.1 g on day 14, 10.8 g vs. 7.9 g on day 21) and heat allodynia (9 s vs. 6 s on day 7, 7 s vs. 4 s on day 14, 8 s vs. 6 s on day 21) on the days 7th, 14th, and 21st post-surgery (*p* < 0.05). Furthermore, there were no significant difference in mechanical and heat perception between the sham group and mice given A-740003 alone.

### 3.2. Inhibition of P2X7R promoted NREM sleep after chronic-constriction injury

**FIGURE 3:**
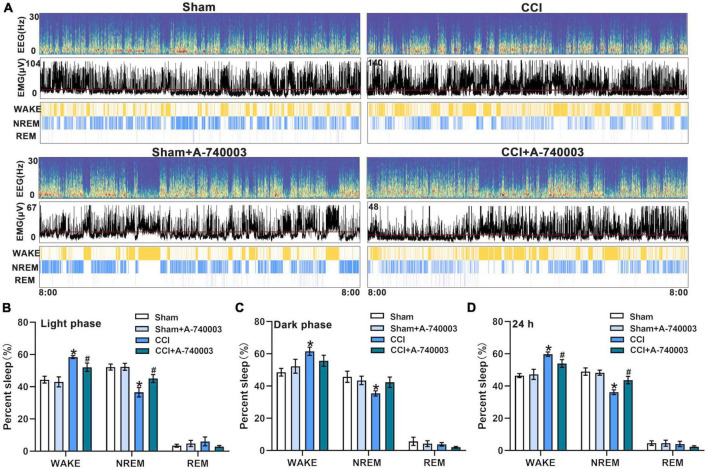
Inhibition of P2X7R promoted NREM sleep after chronic-constriction injury. **(A)** Representative spectrogram of EEG (Top), EMG (Middle), and brain states (Bottom) of four groups during 24 h. **(B–D)** Quantification of the percentage of wake, NREM, and REM sleep states in the light phase **(B)**, dark phase **(C)**, and 24 h **(D)**. Data are presented as mean ± SEM (*n* = 6). *p < 0.05, vs. Sham group, ^#^*p* < 0.05 vs. CCI group. Sham, the Sham group; Sham + A-740003, the Sham mice treated with A-740003; CCI, the chronic-constriction injury group; CCI + A-740003, the CCI mice treated with A-740003.

The EEG and EMG signals were recorded for 24 h as previously described (Seok et al., 2018). The corresponding hypnograms of mice in different groups were examined to confirm the influence of P2X7 receptor on sleep/awake architecture after administration of A-740003 (Figure 3A). When compared to the sham group, mice in the CCI group spent significantly less time in NREM sleep (36.2% vs. 49.0% in 24 h) and more time in wakefulness (59.8% vs. 46.5% in 24 h) during both light and dark phase (*p* < 0.05). The CCI, on the other hand, had no effect on REM sleep state. A-740003 significantly attenuated the decreased NREM sleep time (43.7% vs. 36.2% in 24 h) and increased wake time (54.0% vs. 59.8% in 24 h) in chronic pain mice especially in the light phase (*p* < 0.05). Furthermore, there was no significant difference observed in the states of sleep/wakefulness for giving A-740003, in comparison to the control group (Figures 3B–D).

### 3.3. Inhibition of P2X7R decreased P2X7R-activated microglia in the cortex after chronic-constriction injury

**FIGURE 4:**
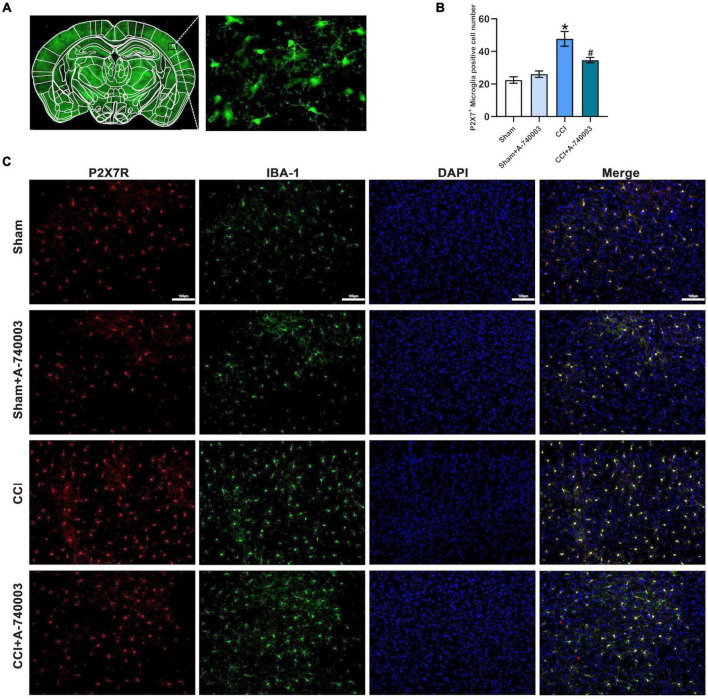
Inhibition of P2X7R decreased P2X7R-activated microglia in the cortex after chronic-constriction injury. **(A)** The expression of microglia in the whole cortex section (Left) and morphology of microglia (Right). **(B)** Quantitative analysis of P2X7R^+^ and Iba1^+^ positive cells in the parietal cortex. **(C)** Immunofluorescence staining of cortex sections in microglial cells in the parietal cortex from mice. P2X7R (red), Iba1 (green), and DAPI (blue). Original magnification: ×20; Scale bar = 100 μm. Data are presented as mean ± SEM (*n* = 3). *p < 0.05, vs. Sham group, ^#^*p* < 0.05 vs. CCI group. Sham, the Sham group; Sham + A-740003, the Sham mice treated with A-740003; CCI, the chronic-constriction injury group; CCI + A-740003, the CCI mice treated with A-740003.

P2X7R were primarily located in microglia, thus the variations of the microglia in the parietal cortex were detected in the current study. Figure 4A showed the expression of microglia in the whole brain and morphology of microglia. The immunohistochemical method for double P2X7R and microglia in the parietal cortex was performed on the 14th day after CCI establishment, and the representative results were depicted in Figure 4C. The number of microglia expressed by P2X7R^+^-Iba1^+^ in CCI mice was significantly higher than in the sham mice (*p* < 0.05). Moreover, the P2X7R inhibitor A-740003 significantly decreased the number of P2X7R^+^ –Iba1^+^ microglia of the CCI group (*p* < 0.05) (Figure 4B). Thus, the results suggested that the expressions of microglial P2X7R could be linked to the chronic pain induced by CCI intervention, which was consistent with former findings (Li et al., 2017).

### 3.4. Inhibition of P2X7R could promote the bias restoration toward faster oscillation in ventral posterior nucleus and primary somatosensory cortex after chronic-constriction injury

**FIGURE 5:**
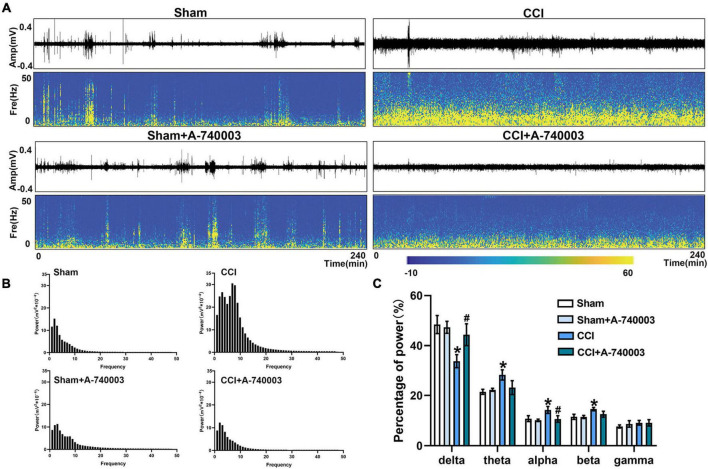
Inhibition of P2X7R could promote the bias restoration toward faster oscillation in ventral posterior nucleus after chronic-constriction injury. **(A)** Representative examples of spontaneous local field potential (LFP) recordings (Top) and spectrograms (Bottom) of the same LFP signals in ventral posterior nucleus of the four groups during 4 h. **(B)** Power spectral histograms of the LFP signals in ventral posterior nucleus. **(C)** Quantitative analysis of relative power percentage within specific frequency bands (delta: 1–4 Hz, theta: 4–8 Hz, alpha: 8–12 Hz, beta: 12–30 Hz, and gamma: 30–100 Hz). Data are presented as mean ± SEM (*n* = 6). *p < 0.05, vs. Sham group, ^#^*p* < 0.05 vs. CCI group. Sham, the Sham group; Sham + A-740003, the Sham mice treated with A-740003; CCI, the chronic-constriction injury group; CCI + A-740003, the CCI mice treated with A-740003.

**FIGURE 6:**
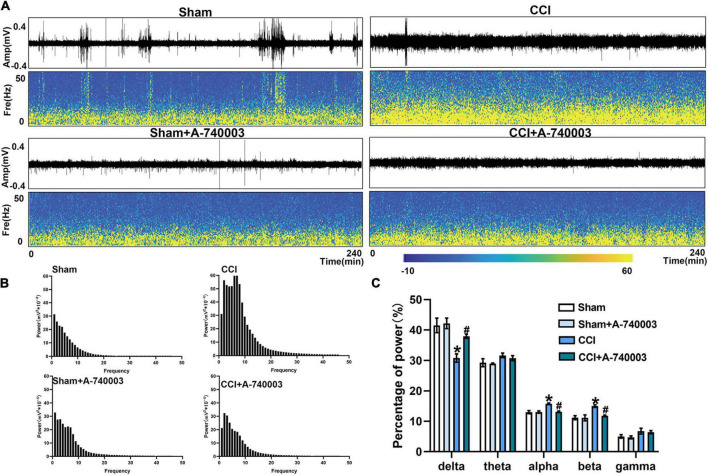
Inhibition of P2X7R could promote the bias restoration toward faster oscillation in primary somatosensory cortex after chronic-constriction injury. **(A)** Representative examples of spontaneous local field potential (LFP) recordings (Top) and spectrograms (Bottom) of the same LFP signals in primary somatosensory cortex of the four groups during 4 h. **(B)** Power spectral histograms of the LFP signals in primary somatosensory cortex. **(C)** Quantitative analysis of relative power percentage within specific frequency bands (delta: 1–4 Hz, theta: 4–8 Hz, alpha: 8–12 Hz, beta: 12–30 Hz, and gamma: 30–100 Hz). Data are presented as mean ± SEM (*n* = 6). *p < 0.05, vs. Sham group, ^#^*p* < 0.05 vs. CCI group. Sham, the Sham group; Sham + A-740003, the Sham mice treated with A-740003; CCI, the chronic-constriction injury group; CCI + A-740003, the CCI mice treated with A-740003.

The spectrograms and power spectral histograms demonstrated the frequency compositions of LFP power spectra in ventral posterior nucleus (VP) (Figures 5A, B) and primary somatosensory cortex (S1) (Figures 6A, B), respectively. The quantitative analysis of the power of these LFP spectra within specific frequency bands were collected in Figures 5C, 6C, such as 1–4 Hz-delta, 4–8 Hz-theta, 8–12 Hz-alpha, 12–30 Hz-beta, 30–100 Hz-gamma. For the regions of VP and S1, the relative power percentage of delta oscillation significantly decreased after the CCI intervention compared to the sham group (33.7% vs. 48.4% in the VP, 30.8% vs. 43.3% in the S1) (*p* < 0.05), which was significantly revised by treated with A-740003 (44.4% vs. 33.7% in the VP, 37.9% vs. 30.8% in the S1) (*p* < 0.05). When compared to the control group, mice with CCI had a higher percentage for the alpha oscillation (14.3% vs. 10.8% in the VP, 15.7% vs. 12.5% in the S1) (*p* < 0.05). However, it was also significantly reversed by adding A-740003 (10.7% vs. 14.3% in the VP, 13.1% vs. 15.7% in the S1) (*p* < 0.05). In contrast, the relative power percentage of beta oscillation was significantly increased after CCI intervention (14.6% vs. 11.6% in the VP, 15.0% vs. 10.4% in the S1) (*p* < 0.05). After A-740003 intervention, these changes were recovered only in S1 (11.8% vs. 15.0%) (*p* < 0.05). CCI intervention also increased the relative power percentage of theta oscillation in VP (28.2% vs. 21.5%) (*p* < 0.05), whereas A-740003 did not reverse the change. Thus, the results demonstrated that the P2X7R inhibition could promote the bias restoration toward faster oscillations.

### 3.5. Inhibition of P2X7R decreased the coherence of local field potential in ventral posterior nucleus and primary somatosensory cortex after chronic-constriction injury

**FIGURE 7:**
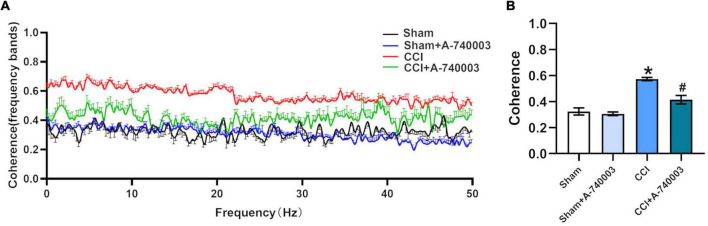
Inhibition of P2X7R decreased the coherence of local field potential in ventral posterior nucleus and primary somatosensory cortex after chronic-constriction injury. **(A)** Coherence of local field potential (LFP) in ventral posterior nucleus (VP) and primary somatosensory cortex (S1) within 1–50 Hz. **(B)** Quantification of the total coherence in the 1–50-Hz range between LFP signals in VP and S1. Data are presented as mean ± SEM (*n* = 5). *p < 0.05, vs. Sham group, ^#^*p* < 0.05 vs. CCI group. Sham, the Sham group; Sham + A-740003, the Sham mice treated with A-740003; CCI, the chronic-constriction injury group; CCI + A-740003, the CCI mice treated with A-740003.

The coherences of LFP (1–50 Hz) for the regions of VP and S1 were analyzed and illustrated in Figure 7A, and they were summarized in Figure 7B. Results revealed that the total coherence significantly increased in the CCI group compared to the sham group (0.57 vs. 0.32) (*p* < 0.05). This variation was significantly reversed by adding the P2X7R inhibitor A-740003 (0.41 vs. 0.57) (*p* < 0.05). These findings suggested that the sleep disruption caused by NP is most likely related to coherence changes between VP and S1, which can be reversed by P2X7R.

### 3.6. Inhibition of P2X7R ameliorated increased glutamate level after chronic-constriction injury

**FIGURE 8:**
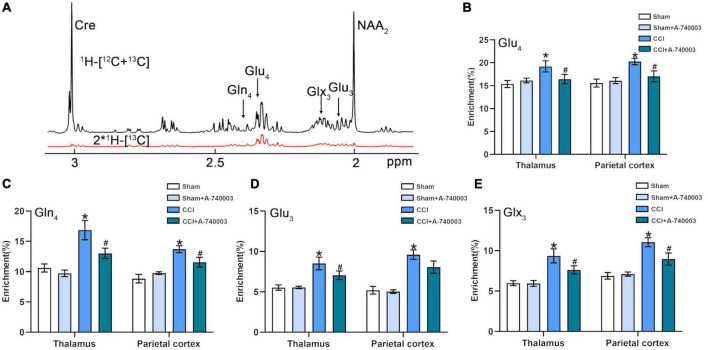
Inhibition of P2X7R ameliorated increased glutamate level after chronic-constriction injury. **(A)** Representative ^1^H-[^13^C]-NMR spectra obtained from thalamus. The top spectra (black) represented the total enrichment ^1^H-[^12^C + ^13^C], whereas the bottom one (red) represented ^13^C labeling of metabolites 2*^1^H-[^13^C]. Asp, aspartate; Cre, creatine; Gln, glutamine; Glu, glutamate; Glx, glutamine + glutamate; NAA, N-acetylaspartate; Subscript number, ^13^C labeled positions in different metabolites. **(B–E)** Quantification of enrichment of Glu_4_ **(B)**, Gln_4_ **(C)**, Glu_3_ **(D)**, Glx_3_ **(E)** in thalamus and parietal cortex for the four groups. Data are presented as mean ± SEM (*n* = 8). *p < 0.05, vs. Sham group, ^#^*p* < 0.05 vs. CCI group. Sham, the Sham group; Sham + A-740003, the Sham mice treated with A-740003; CCI, the chronic-constriction injury group; CCI + A-740003, the CCI mice treated with A-740003.

In the current study, the [1-^13^C] glucose metabolism method based on NMR detection was applied to evaluate the variations of cerebral metabolic kinetics in the regions of the thalamus (TH) and parietal cortex (PC). The representative NMR spectrum from TH is illustrated in Figure 8A, and the related metabolites were labeled. Compared to the control group, the metabolism of CCI group showed increased ^13^C enrichments for Glu_4_ (19.2% vs. 15.4% in the TH; 20.3% vs. 15.6% in the PC), Gln_4_ (16.9% vs. 10.6% in the TH; 13.7% vs. 8.8% in the PC), Glu_3_ (8.5% vs. 5.5% in the TH; 9.6% vs. 5.2% in the PC) and Glx_3_ (9.4% vs. 6.0% in the TH; 11.1% vs. 6.9% in the PC) in both regions (*p* < 0.05). After A-740003 intervention, some changes were recovered, including Glu_4_ (16.4% vs. 19.2%), Gln_4_ (13.0% vs. 16.9%), Glu_3_ (7.1% vs. 8.5%), and Glx_3_ (7.6% vs. 9.4%) in the TH and Glu_4_ (17.0% vs. 20.3%), Gln_4_ (11.6% vs. 13.7%), and Glx_3_ (9.0% vs. 11.1%) in the PC (*p* < 0.05) (Figures 8B–E). Thus, the results demonstrated that the P2X7R inhibition could promote the restoration of most disturbed metabolites after the CCI intervention, which is probably also related to the CCI-induced sleep disorder.

## 4. Discussion

Sleep disturbances frequently occur in patients suffering from chronic pain. Moreover, it can contribute to next-day sleepiness and fatigue, impairing the cognitive function (Marshansky et al., 2018). However, the mechanisms underlying NP-induced sleep disturbance remain unknown. Analyzing pain-sleep interaction factors could thus improve the clinical status of patients suffering from sleep disorders and pain.

Herein, we evaluated the effects of P2X7R on sleep disturbance induced by NP using the CCI model mice. We have analyzed the pain-related behaviors by MWT and PWL, and the results were consistent with previous studies, a stable hypersensitivity to pain was discovered compared to the sham group after the CCI establishment (Jiang et al., 2021). It has been demonstrated that NP was probably related to the structures and functions of VP and S1, where integrated pain sensory, emotional, sleep, and attentional components (Kim et al., 2017; Asseyer et al., 2020). Furthermore, various studies have shown that cortical microglia were identified as principal elements to pain, neuronal survival, synaptic pruning, sleep disturbances, and neurotransmitter release (Badimon et al., 2020; Iovino et al., 2020; Liu et al., 2021; Cornell et al., 2022). Microglial activation is in the hope of developing new therapeutics for treating neurodegenerative diseases and chronic pain (Carniglia et al., 2017). Recently, P2X7R has received greater attention due to its role in neurological diseases, such as pain, sleep disturbance, and cognition disorder, and the effective role of P2X7R comprehends neuroinflammation, neuroglia, and neurotransmitters (Jimenez-Mateos et al., 2019; Chen et al., 2021; Zhang et al., 2022). Moreover, it has been reported that in the brain, P2X7R is an ionotropic receptor widely presented in glial cells and neurons (Bernal-Chico et al., 2020), and located predominantly on microglia (Yue et al., 2017). P2X7R plays an important role in microglial activation responses. The concurrent increases in the expression of P2X7R and activation of microglia suggest that P2X7R-induced microglial activation may contribute to the microglial function (Fernandes et al., 2016). In our study, we found that CCI increased microglial P2X7R expression in the cortex, different from sham mice. Additionally, administration of P2X7R inhibitor recovered these changes. The dependence of the negative association between CCI-induced pain related behaviors and NP on the presence of microglial P2X7R observed in this study suggests that microglial P2X7R may be essential and could function as a regulator of NP.

Furthermore, we discovered abnormalities in the sleep architecture of mice with peripheral nerve injury. Wakefulness was reduced and fragmented, indicating that CCI mice are unable to maintain wakefulness in a normal manner. Our observations were consistent with previously reported sleep disturbance in NP mice (Ito et al., 2022) and the critical roles of the P2X7R in sleep disturbance induced by NP (Krueger et al., 2010). Notably, the administration of P2X7R inhibitor could effectively recover the activation of microglia in cortex, the decrease of NREM sleep and the increase of wakefulness in CCI mice during the 24-h cycle. Increasing evidence has demonstrated that almost complete microglial depletion impaired excitatory neurotransmission and enhanced the duration of NREM sleep (Corsi et al., 2022), which might contribute to sleep disturbance in chronic NP. Indeed, recent research demonstrated that cortical microglia modulated neuronal activity in NREM sleep duration and presented diurnal variation in spatial area and structural complexity (Hayashi et al., 2013). The diurnal variation of extracellular ATP concentration was identified as an essential factor why cortical microglia played a key role in the sleep-awake cycle (Hayashi et al., 2013). Therefore, as a ligand-gated ion channel dependent on ATP, microglial P2X7R plays an important role in sleep disorders. After an injury, reactive microglia increased in cortex regions, preceding the development of sleep disruptions (Bellesi et al., 2017). Given the probable causal relationship between pain relief and sleep disorder improvement induced by administration of P2X7R inhibitor, microglial P2X7R expression changes might be a response to pain relief and subsequently improved sleep. Further, the data confirmed the relief effects on sleep disturbance of P2X7R, which might conduce to exploration of the causal mechanisms.

Neural oscillations are ended up causing by the rhythmic activity of neuron clusters. In animal models, the relationship between sleep disorders induced by chronic pain and network oscillations has attracted much attention in recent years (Adamantidis et al., 2019). Herein, we recorded local field potentials (LFP) within the VP and S1 in freely moving mice under spontaneous pain conditions. The LFP oscillations were coordinated between thalamus and cortex in a manner peculiar to the sleep phase, and abundant data have indicated that pain contributed to sensitization of neurons at the single unit level in VP (Asseyer et al., 2020) and S1 (Okada et al., 2021). The rodents take turns into the NREM sleep characterized by slow oscillations, delta waves (1–4 Hz), and spindles in the scalp EEG or intracranial LFPs, whereas the REM stage predominantly consists of theta (4–8 Hz) rhythm (Vantomme et al., 2019; Uygun and Basheer, 2022). Cortical cells, on the other hand, fire tonically during aroused states, and the oscillations are dominated by low-amplitude fast activity in the beta (12–30 Hz) and gamma (30–100 Hz) frequency ranges (Cheron et al., 2016). The delta rhythm (1–4 Hz) has long been thought to be strongly connected with sleep duration and intensity, the alpha oscillation (8–12 Hz) has been increasingly regarded as reflecting the brain’s spontaneous activity in the relaxed eyes-closed state, and the beta oscillations (12–30 Hz) have been recorded in quietly sitting normal animals when they are in a state of awakening (Cheron et al., 2016). In our study, chronic pain decreases the power of low-frequency oscillations (1–4 Hz) while increasing the power of high-frequency oscillations (8–30 Hz) localized to S1 and VP, demonstrating CCI-induced sleep disorder. P2X7R is an ATP-gated ionotropic channel implicated in sleep regulation and as well as the regulation of various neuronal functions, such as neurotransmitter release and synaptic transmission, and contributing to the formation of network oscillations (Jimenez-Mateos et al., 2019; Ribeiro et al., 2019). ATP is released with neuro- and glio-transmission, and neuronal activity is greater during wakefulness (Dworak et al., 2010). Thus, inhibiting P2X7R leads to decreased wakefulness, consistent with our research. These neural oscillations indicated that increased wakefulness and decreased NREM occurred in CCI, and P2X7R in microglia contributed the sleep disturbances induced by NP.

Thalamocortical oscillations are defined as rhythmic activity generated by thalamocortical connections necessary for sensory perception processing (Adamantidis et al., 2019). The neurons of S1 received dense projections from the VP, a primary target of ascending sensory tracts in the spinal cord and brainstem (Killackey and Sherman, 2003). Previous studies have demonstrated that cortically induced delta oscillation and slow wave might be reflected in the thalamus, which was elicited by network properties of thalamic and thalamocortical neurons (Steriade et al., 1993). Although pain has a strong influence on synchrony in thalamocortical oscillations (LeBlanc et al., 2014), the effects of sleep disruption are debatable. Painful sensory experiences, as expected, generated sleep disturbance by modulating thalamocortical oscillations. Enhanced thalamocortical coherence has been reported in NP patients (Sarnthein and Jeanmonod, 2008), similar to these observations, our results indicated that total thalamocortical coherence between 1 and 50 Hz augmented in our model. The pooled coherence of thalamocortical circuit oscillations was altered during the NREM sleep, indicating that the connectivity of the thalamocortical circuitry was related to sleep (Tsai et al., 2010). It has been previously reported that thalamocortical functional connectivity (FC) decreased during the period of transition from wakefulness into sleep (Hale et al., 2016). Similarly, we discovered that increased thalamocortical coherence between 1 and 50 Hz, it might result in decreased NREM sleep. Furthermore, increased extracellular concentrations of ATP accelerated the activity of the subplate neurons, which is linked to functional thalamocortical connections (Beamer et al., 2017). Hence, inhibiting P2X7R leads to decreased thalamocortical coherence, which is consistent with our research. These results indicated that P2X7R disturbed thalamocortical connections, contributing to the NP-induced sleep disorder.

Glutamate (Glu) is the primary excitatory neurotransmitters in the central nervous system (CNS) (Barros-Barbosa et al., 2018; Ju and Tam, 2022). It has been suggested that the regulation of these amino acids performs an important role in the sleep-wake cycle of normal brain activity (Jones, 2020), which may play a significant neural modulation role in the neural activity (Xu et al., 2021). In the glutamate-glutamine cycle, glutamate released from the nerve terminals, then was absorbed by the surrounding glial cells and returned to the nerve terminals in the form of glutamine, which played the role of neurotransmitters and participated in neuroregulation (Rothman et al., 1999). Recently, P2X7R was discovered to be expressed in the thalamus and cortical nerve terminals of mice with chronic NP (Jimenez-Mateos et al., 2019). Previous studies have shown that with activated P2X7R, glutamate release consistently increased, and exceeded GABA release from nerve terminals in the cortex (Barros-Barbosa et al., 2018). Proper regulation of these neurotransmitters was essential for sleep, and thus mechanisms of pain-induced sleep disturbance might be associated with changes in neurotransmitter imbalance (Jones, 2020). Glutamate, mediated via excitatory synaptic transmission, can modulate the neuronal activity in the brain’s neuronal networks (Minoshima et al., 2020). Moreover, cellular responses were triggered by the release of glutamate and GABA, including increasing cell proliferation, releasing cytokines and regulating neuronal excitability, when ATP bound to P2X7R (Scemes and Velíšková, 2019). Here, we demonstrated that chronic NP activated the expression of microglial P2X7R, resulting in increased levels of Glu released from TH and PC. P2X7R activation induced glutamate efflux in nerve terminals of the cerebral cortex, depending on extracellular Ca^2+^ concentrations (Alloisio et al., 2008). T-type calcium channels were essential for burst discharges of thalamic neurons, which affected the sleep-awakening transition altering delta oscillations (1–4 Hz) and sleep spindles (7–15 Hz) (Lee and Shin, 2007). Extracellular Glu levels were elevated when aroused from sleep, peaked during the transition from awakening to NREM sleep, and decreased during NREM sleep (Azuma et al., 1996). Therefore, Glu levels in the neurons of the thalamus and cortex might contribute to sleep disturbance induced by NP, demonstrating that P2X7R was involved in sleep disturbance induced by NP through the modulation of neurotransmitters.

## Conclusion

Our findings suggested that peripheral nerve injury could activate the microglial P2X7R in the parietal cortex. A significant variation of the sleeping structures was detected following the treatment of peripheral nerve injury, which was reversed by the P2X7R inhibitors. The investigations of the LFP in the regions of VP and S1 revealed that the peripheral nerve injury could change the power and coherence of the LFP in both regions, and the metabolic kinetics study also revealed that most metabolites were also changed after the treatment of peripheral nerve injury. The majority of these changes were significantly reversed by P2X7R inhibitor. The variations of the LFP and metabolic kinetics probably related to the sleep disorders induced by the peripheral nerve injury, which could provide some therapeutic basis for the clinical treatment of sleep disorders by peripheral nerve injury or peripheral nerve injury itself.

## Data availability statement

The raw data supporting the conclusions of this article will be made available by the authors, without undue reservation.

## Ethics statement

This animal study was reviewed and approved by the Experimental Animal Welfare Ethics Committee of Zhongnan Hospital of Wuhan University (No: ZN2021212).

## Author contributions

TL and YG: methodology. MH and ZG: formal analysis. BZ and JZ: investigation. TL: writing—original draft preparation. JW: writing—review and editing. YC and TC: data curation. QZ: supervision. ZZ: funding acquisition. All authors have read and agreed to the published version of the manuscript.
